# Breast and cervical cancer survival at Instituto Nacional de Cancerología, Colombia

**DOI:** 10.25100/cm.v49i1.2840

**Published:** 2018-03-30

**Authors:** Constanza Pardo, Esther de Vries

**Affiliations:** 1 Cancer Surveillance Group, Instituto Nacional de Cancerología, Bogota, Colombia; 2 Department of Clinical Epidemiology and Biostatistics, Pontificia Universidad Javeriana, Bogota, Colombia

**Keywords:** Breast cancer, cancer of the uterine cervix, survival analysis, hospital-based, registry, Colombia, cáncer de mama, cáncer de cuello uterino, análisis de supervivencia, registro hospitalario de cáncer, Colombia

## Abstract

**Objective:**

to provide and compare estimations of two-year overall survival for cervical and female breast cancer in three cohorts (first treated in 2007, 2010, 2012) at the Instituto Nacional de Cancerología of Colombia

**Methods:**

All patients first treated at the Instituto Nacional de Cancerología for breast or cervical cancer in the years 2007, 2010, 2012, without a prior cancer diagnosis, were included for the study. The hospital-based cancer registry was cross linked with governmental databases to obtain follow-up information on all patients. Probability of surviving 24 months since the date of entry at the hospital was estimated using Kaplan-Meier methods, using the log-rank test to evaluate differences between groups.

**Results:**

We analyzed 1,928 breast cancer cases and 1,189 cervical cancer cases, resulting in an overall survival probability at 24 months of 79.6% (95% CI: 77.8-81.4) for BC and of 63.3% (95% CI: 60.6- 66.0) for cervical cancer, there were no differences in survival for year of entry. Advanced clinical stage substantially affected overall survival, being 32.2% (95% CI: 28.4-44.0) for stage IV breast cancer and 22.6% (95% CI: 11.4-33.8) for stage IV cervical cancer.

**Conclusions:**

Breast cancer was the cancer with the best survival rates at Instituto Nacional de Cancerología; cervical cancer was the one with the lowest survival rates. Overall survival did not change over the years for any of the cancers.

## Resumen

**Objetivo:**

Describir las estimaciones de supervivencia global a dos años para mama (mujeres) y cuello uterino en tres cohortes (tratadas por primera vez en 2007, 2010, 2012) en el Instituto Nacional de Cancerología de Colombia.

**Métodos:**

Se incluyeron las pacientes tratadas por primera vez en el Instituto Nacional de Cancerología por cáncer de mama y de cuello uterino en los años 2007, 2010, y 2012, y quienes no habían tenido un diagnóstico previo de otro cáncer. Se cruzaron las bases de datos del registro hospitalario de cáncer con las gubernamentales para obtener información de seguimiento de los casos. Se estimó la probabilidad de sobrevivir a 24 meses a partir de la fecha de ingreso mediante el método de Kaplan-Meier. Se aplicó la prueba de rango logarítmico para evaluar las diferencias entre los grupos.

**Resultados:**

Se analizaron 1,928 casos de cáncer de mama y 1,189 de cuello uterino. La estimación de la supervivencia global a 24 meses para mama fue 79.6% (IC 95%: 77.8-81.4) y de 63.3% (IC 95%: 60.6-66.0) para cuello uterino, no se observaron tendencias en supervivencia con el año de ingreso. En los estadios clínicos avanzados la supervivencia global disminuyó en estadio clínico IV, tanto para cáncer de mama, 32.2% (IC 95%: 28.4-44.0), como para cuello uterino 22.6% (IC 95%: 11.4-33.8).

**Conclusiones:**

El cáncer de mama presentó mejor supervivencia en el Instituto Nacional de Cancerología frente al cáncer de cuello uterino. La supervivencia global se comportó de manera estable con los años para ambos tipos de cáncer.

## Introduction

The cancer burden among women in Latin-America is mostly attributable to breast and cervical cancers ^1^ ^-^ ^3^. In 2012 in Latin-America, age-adjusted breast cancer incidence rates reached levels between 40-65 per 100,000 woman-years. Age-standardized breast cancer mortality rates in Colombia increased substantially: from 6.9 in the mid-1980´s (6.9) to 10.8 in 2012 ^4^ ^-^ ^6^. Age-standardized cervical cancer mortality rates have decreased from 13.2 in 1984-1988 to 8.7 in 2013 ^6^. 

The Instituto Nacional de Cancerología of Colombia (INC) estimated that in the period 2007-2011, around 7,600 new breast cancer cases were diagnosed annually, with 2,226 annual breast cancer deaths. In the same period, there were 4,462 new cervical cancer cases, and 1861 deaths. Both cancer types show strong geographical variations between different parts of the country, with breast cancer being more frequent in the cities and urbanized areas, and cervical cancer in the more remote areas ^7^.

The prognosis of these two cancer types depends on sociodemographic characteristics but even more on the stage at diagnosis, the available therapeutic options and the efficiency of the system in providing (access to) care. In general, cancer survival improves with Human Development Index (HDI) of countries or regions, probably through better access to efficient treatments and potentially early detection. Colombia is currently categorized as a high HDI country (0.720) ^8^. Since 2003 the coverage of Colombia's ‘universal’ mandatory health insurance system has increased substantially. This system consists mainly of two different regimes, each covering slightly under 50% of the population, in which people are assigned on the basis of income: the contributory regime, covering workers and their families with an income above the cut-off and financed through the payroll and employer's contributions and the subsidized regime, covering those identified as ‘poor’. Additionally, around 5% of the population, workers in the petrol industry, teachers, military and police, is affiliated to “special” and “exceptional” regimes; and there is a remaining group of the population which is not covered by the system (representing 2.6% in 2015, according to the Ministry of Health) ^9^ ^,^ ^10^. The insurance packages and methods are similar, but not equal between regimes and providers within each regime. 

There is little available data on survival of these cancer types in Latin American populations; the little population-based data does not show survival by stage ^11^ ^,^ ^12^. Trends in survival in hospital-based settings are scarce ^13^, with most existing reports aiming to determine the efficiency of the different therapeutic options. INC Colombia designed a survival surveillance system, based on linkage with government databases, to produce comparable overall survival estimates of its patients on an annual basis, with the objective of evaluating changes in prognosis over time and contributing to the improvement of the quality of cancer care within the institution and, through comparison of data, at a national level.

In this manuscript we compare the demographical and clinical characteristics of breast and cervical cancer patients treated at INC in the years 2007, 2010 and 2012 and report 2-year overall survival estimated by age, clinical stage and type of affiliation to the social security system.

## Materials and Methods

All female invasive breast (C50) and cervical cancer (C53) cases first treated at the Colombian INC in the years 2007, 2010 and 2012 were selected from the hospital-based cancer registry of the INC ^14^. Only the first primary invasive cancer was considered for each cancer, since the probability of survival of patients with previous primaries may be altered. The cases registered during each year were considered fixed cohorts. The hospital registry data was checked and completed using medical records and linked with the hospital-based mortality database as well as government-based information sources such as the National Civil Registry (Registraduría Nacional del Estado Civil (RNEC)) to determine vital status on December 31^st^ 2014 and date of death for deceased patients who died extramurally. This was a necessary step since the Colombian legislation does not allow direct linkage between outpatient databases and the cause- and date of death registry; if one has a personal identification number, it is possible to check for vital status and reporting of deaths in RNEC. Two-year overall survival was calculated for the cohorts of women entering INC in 2007, 2010 and 2012, with start date of follow-up being the date of entry at INC. Date of death was specified according to the death certificate in case this certificate was available, for those patients reported as deceased in the RNEC but without detailed date of death, we determined the expected date of death as the date of reported deceased at RNEC minus a correction factor. This correction was calculated based on data of deceased patients with available death certificates, where the real date of death was compared with the date of reporting in RNEC; the median difference between these dates was subtracted from the RNEC date to obtain expected date of death ^15^. This median number of days of difference between date of death and reporting of the death decreased over time (for breast cancer it was 148 days in 2007, 66 in 2010 and 25 in 2012; corresponding number of days for cervical cancer was 184, 114 and 39 days), indicating substantial improvements in the reporting systems. The detailed steps to determine date of last contact or date of death are described in detail elsewhere, and summarized below ^15^.

Since this process is a bit complex, we describe it in more detail below: 

a) For patients who died within INC, the exact date of death was known and assigned.

b) For patients with unknown vital status, we used the Colombian personal identification number (cédula) to check for vital status in the databases of the RNEC - RNEC reports if persons are deceased. If the patients did not appear as “deceased” in any of the RNEC data sources, the 31st of December 2014 was assigned at date of last follow-up. 

c) For those cases reported as deceased in RNEC but without death certificate information, the date of death was estimated based on the date of reporting of the death in RNEC, corrected by the median difference between date of death and date of reporting of death at RNEC, as described above and in detail elsewhere ^15^. If this procedure resulted in negative survival times, the date reported in RNEC was assigned as date of death. This procedure generated the variable: calculated date of death.

d) For those cases deceased according to RNEC but with only year of death known (no month or day available in RNEC), we assigned the 30th of June of the provided year as date of death for patients with date of entry in the first semester of a year, and 31st of December if patients entered INC in the second semester of a year.

e) For those cases in which none of these methods could be applied, or which were not identified in the mentioned databases, the last date of follow-up was assigned as the date of the last visit according to the medical file at INC. 

### Statistical analysis

In order to assess differences in distribution of clinical stage by type of affiliation to the Colombian social security system, we performed Fishers exact test. Survival time was calculated as the difference between the closing date of follow-up (December 31^st^, 2014), date of last contact or calculated date of death and the date of entry at the INC. The probability of surviving 24 months was calculated using Kaplan-Meier analysis, and differences in survival by several variables was assessed using the log-rank test. Univariate analyses were performed for year of entry, age in two categories (<50 y ≥50 years), clinical stage and type of affiliation to the social security system at the moment of entry at INC. Because of violation of the proportional hazard assumption for the variable years, age group, and type of affiliation to the social security system, we did not report results of the multivariate Cox Proportional Hazards models. All data was analyzed using SPSS®, v19.

## Results

### Demographic and clinical characteristics of the patients

We analyzed a total of 1,928 breast and 1,189 cervical cancer patients. Table 1 shows the characteristics by cancer type and cohort; the distribution between breast and cervical cancer was shifting towards breast cancer over time. Breast cancer cases were concentrated in the 45-54 years age group, and cervical cancer in the 15-44 years age group. The percentage of patients not affiliated to the social security system decreased between 2007 and 2012 for both cancer types. There were an important proportion of cases without clinical stage information (19.7% for breast cancer, 15.0% for cervical cancer); 100% of cases had a histologically confirmed diagnosis. 

**Table 1:** Demographic and diagnostic characteristic of the study populationby cohorts

Characteristics	Breast cancer								Cervical cancer							
	n	%	2007		2010		2012		n	%	2007		2010		2012	
Total number	1,928	100	622	32.3	632	32.8	674	35.0	1,189	100	387	32.5	474	39.9	328	27.6
Age (years)	*** ***	*** ***														
0 - 14	0	0.0	0	0.0	0	0.0	0	0.0	0	0.0	0	0.0	0	0.0	0	0.0
15 - 44	344	17.8	118	19.0	102	16.1	124	18.4	400	33.6	119	30.7	167	35.2	114	34.8
45 - 54	609	31.6	189	30.4	199	31.5	221	32.8	293	24.6	102	26.4	97	20.5	94	28.7
55 - 64	493	25.6	158	25.4	165	26.1	170	25.2	262	22.0	86	22.2	116	24.5	60	18.3
> 65	482	25.0	157	25.2	166	26.3	159	23.6	234	19.7	80	20.7	94	19.8	60	18.3
Social Security Scheme	*** ***	*** ***														
Contributive	764	39.6	228	36.7	236	37.3	300	44.5	286	24.1	86	22.2	85	17.9	115	35.1
Subsidized	525	27.2	119	19.1	219	34.7	187	27.7	573	48.2	154	39.8	289	61.0	130	39.6
Special	168	8.7	50	8.0	50	7.9	68	10.1	45	3.8	9	2.3	9	1.9	27	8.2
Particular	256	13.3	90	14.5	80	12.7	86	12.8	98	8.2	30	7.8	39	8.2	29	8.8
Uninsured	215	11.2	135	21.7	47	7.4	33	4.9	187	15.7	108	27.9	52	11.0	27	8.2
Clinical stage	*** ***	*** ***														
I	111	5.8	26	4.2	43	6.8	42	6.2	289	24.3	106	27.4	112	23.6	71	21.6
II	545	28.3	168	27.0	196	31.0	181	26.9	250	21.0	92	23.8	104	21.9	54	16.5
III	751	39.0	259	41.6	248	39.2	244	36.2	418	35.2	121	31.3	173	36.5	124	37.8
IV	141	7.3	34	5.5	54	8.5	53	7.9	54	4.5	14	3.6	21	4.4	19	5.8
No information	380	19.7	135	21.7	91	14.4	154	22.8	178	15.0	54	14.0	64	13.5	60	18.3

### Overall survival

**Figure 1:**
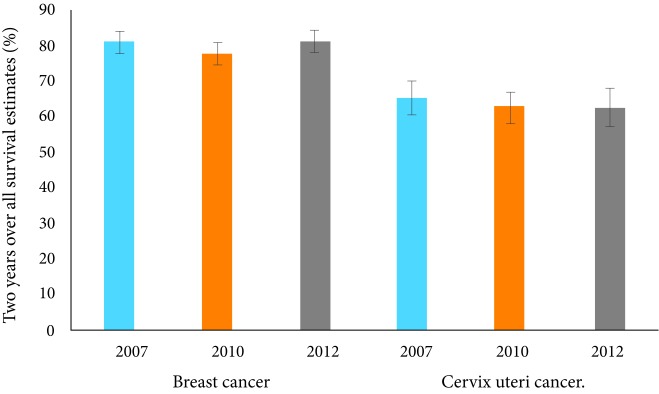
Comparison of two year overall survival estimates for breast and cervical cancer by cohort

Two-year overall survival did not vary between the different cohorts, as shown in Figure 1. 

### Breast cancer

The 1928 patients in the analyses had a median age at entry in INC of 55 years (range 17-99). Most (66%) patients were aged over 50 and most of them (39.6%) were affiliated in the “contributive” regime of the social security system. About half of the patients had stage III-IV breast cancer at entry in INC. Stage distribution differed substantially and statistically significantly between regimes, with around 60.0% of women in the subsidized and uninsured groups being diagnosed in stages III-IV, versus 42.0% in the contributive and around 30.0% in the special regime and privately insured group (Table 2). 

**Table 2:** Distribution of cancer stage by type of affiliation to the social security system

Tumour stage	Contributive	Subsidized	Uninsured	Special	Private
	%	%	%	%	%
Breast cancer stage					
I	8.2	2.1	2.3	12.5	4.3
II	32.1	25.1	22.3	35.8	23.4
III	35.0	51.1	48.8	29.2	24.2
IV	6.9	8.6	13.5	3.0	3.5
No information	17.8	13.1	13.0	19.6	44.5
Cervical cancer stage					
I	26.9	25.2	20.3	31.1	16.3
II	21.7	21.1	21.4	20.0	18.3
III	33.5	35.7	42.8	24.4	26.5
IV	5.2	4.4	5.4	2.2	3.0
No information	12.6	13.6	10.2	22.2	35.7

*p*-values for differences in stage distribution by regimen: Breast cancer: *p*= 0.01034, cervical cancer *p* <0.005

**Figure 2:**
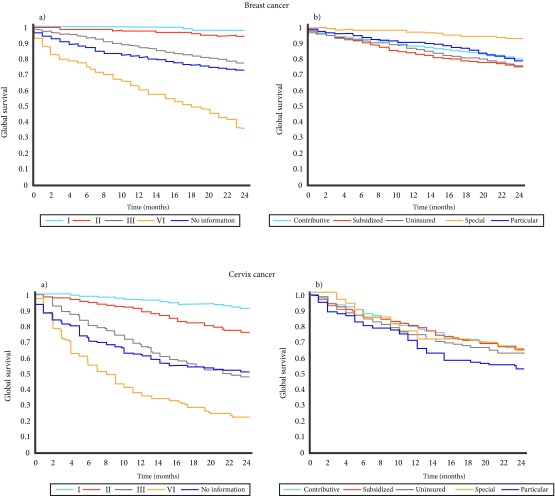
Two-year survival function (Kaplan - Meier) by clinical stage and social security for breast (a and b) and cervical cancer (c and d).

At 24 months of follow-up, 393 (20.4%) had died, the remaining 1,535 cases were censored at follow-up. Two-year OS for breast cancer was 79.6%, with clear differences in survival between types of affiliation to the social security system, being highest for those in the “special” regime (93.4%) (log-rank test 48.9, *p* <0.001). Few patients died in the first month of follow-up (1.9%). Two-year OS of stage I patients was very high (98.2%), declining to 36.2% for stage IV patients. No significant effects were found for year of or age at entry at INC (Table 3, Fig. 2). 

**Table 3:** Univariate overall survival estimates of breast cancer and cervical cancer by cohorts

	Breast cancer						Cervical cancer					
Characteristics	n	%	Number of deaths*	Surviving**	CI 95%	Log-rank test	n	%	Number of deaths*	Surviving**	CI 95%	Log-rank test
Total	1,928		393	79.6	77.8 - 81.4	N.A.	1,189		435	63.3	60.6 - 66.0	*N.A.*
Years of entry at INC												
2007	622	32.3	122	80.4	77.3 - 83.5	X^2^= 4.1	387	32.5	135	65.0	60.3 - 69.7	X^2^= 3.7
2010	632	32.8	142	77.4	74.1 - 80.7	*p=* 0.127	474	39.9	177	62.6	58.3 - 66.9	*p=* 0.161
2012	674	35.0	129	80.8	77.9 - 83.7	* *	328	27.6	123	62.3	57.0 - 67.6	* *
Age (years)						* *						* *
<50	655	34.0	138	78.9	75.8 - 82.0	X^2^= 0.5	539	45.3	173	67.8	63.9 - 71.7	X^2^= 17.6
≥ 50	1273	66.0	255	80.0	77.8 - 82.2	*p=* 0.481	650	54.7	262	59.5	55.8 - 63.2	*p=* 0.000
Social Security Scheme												
Contributive	764	39.6	147	80.8	78.1 - 83.5	X^2^= 48.9	286	24.1	102	64.2	58.7 - 69.7	X^2^=6.0
Subsidized	525	27.2	131	75.0	71.3 - 78.7	*p=* 0.000	573	48.2	201	64.8	60.9 - 68.7	*p=* 0.202
Special	168	8.7	11	93.4	89.7-97.1	* *	45	3.8	16	64.4	50.5 - 78.3	* *
Particular	256	13.3	52	79.6	74.7 - 84.5	* *	98	8.2	46	52.6	42.6 - 62.6	* *
Uninsured	215	11.2	52	75.8	70.1 - 81.5	* *	187	15.7	70	62.4	55.3 - 69.5	* *
Clinical stage												
I	111	5.8	2	98.2	95.6 - 100.7	X^2^= 404.9	289	24.3	28	90.3	87.0 - 93.6	X^2^= 229.5
II	545	28.3	30	94.5	92.5 - 96.5	*p=* 0.000	250	21.0	61	75.6	70.3 - 80.9	*p=* 0.000
III	751	39.0	171	77.2	74.3 - 80.1	* *	418	35.2	217	47.6	42.7 - 52.5	* *
IV	141	7.3	90	36.2	28.4 - 44.0	* *	54	4.5	41	22.6	11.4 - 33.8	
No information	380	19.7	100	73.6	69.1 - 78.1		178	15.0	88	50.6	43.3 - 57.8	

*Number of deaths in two years follow up

**Probability of surviving 2 years

N. A.= not applicable

### Cervical cancer

The median age of the 1,189 women with cervical cancer was 51 years (range 19-92). Most were aged over 50, almost half (48.2%) were affiliated to the subsidized part of the social security system, and almost 50.0% of patients presented at INC with stage I or II disease. The distribution by stage was similar between regimes, with between 40 and 50.0% of women being diagnosed in stages I-II, with the exception of the privately insured women (34.6%), who had a significantly better stage at entry (Table 2). At the end of the two years of follow-up, 435 (36.6%) had died, the remaining 754 cases were censored. The probability of surviving two years was 63.3%, with a better survival for women presented at younger ages (67.8%). During the first month of follow-up, 2.3% of patients died. There was a sharp gradient in survival by clinical stage at presentation, between 90.3% in stage I and 22.6% in stage IV. No differences in survival were observed by year or type of social security (Table 3, Fig. 2). 

## Discussion

These results are among the first of the overall survival surveillance project of the INC; showing a stable two-year overall survival for breast and cervical cancer with the expected differences by clinical stage and important differences by type of social security affiliation for breast cancer between 2007 and 2012. The well-known association between socioeconomic indicators and breast and cervical cancer are reflected in the higher proportion of breast cancer patients in the contributive and special regimes, versus the majority of cervical cancer being affiliated to the subsidized regime, while the population-distribution of affiliated persons does not differ much between the two regimes ^16^. 

There are very few publications on survival of breast and cervical cancer in Colombia and Latin America ^11^ ^,^ ^12^ ^,^ ^17^. Our reported overall 2-year survival for breast (80%) and cervical cancer (63%) is not far from the population-based reports from Cali (3-year OS:breast 77%, cervix 63%) ^12^. Survival in a specialized cancer hospital is expected to be a bit lower than the population-based survival data, because specialized centers tend to receive “complicated” patients referred from other, less specialized hospitals. 

One limitation of our study is that we have no reliable incidence date, and therefore we had to use date of entry at the INC. The survival time calculated from the date of diagnosis is certainly higher than our reported survival data, although we cannot know how much higher: some patients come for their initial treatment to the INC - their date of entry will be close to their date of diagnosis; others come when initial treatment has failed or when they have a recurrence, sometimes a long time after their initial diagnosis. Considering this, our reported survival rates for cervical cancer are relatively similar to European estimates, lower than of the USA but higher than those for Brazil ^11^ ^,^ ^12^. Breast cancer survival is quite a bit lower than reports from USA-based studies, undoubtedly related to a relatively late stage at entry in our patients, with 46% of women with stage III/IV disease. 

The quality of the Colombian death registry has improved substantially in recent years ^18^, limiting, but not eliminating, the possibility that some patients may have died without being registered. In this case, the patient will have been censored alive at their last visit in INC, which may have slightly over-estimated survival rates. The improved quality is also reflected in the diminishing of the time between date of death and reporting of death in RNEC.

The age distribution of our patients was as expected ^19^ ^,^ ^20^. For the prognosis of breast cancer, early detection is important, as well as the time between first symptoms or abnormal screening test and first consultation (according to a previous Colombian study this was >1 month in 34.1% of the patients) and the time between the first consultation and treatment initiation (in 69.8% of patients >3 months in a previous study) ^21^. Cervical cancer patients were, as expected, relatively young, and a substantial proportion (40%) presented with late stage disease, a situation that could have been prevented by effective screening programs. 

Unfortunately, we did not have information on clinical stage for an important proportion of our patients (breast cancer 19.7% and cervical cancer 15.0%). However, our observations of around 50% of women being diagnosed in stage III/IV breast cancer and 40% in stage III/IV cervical cancer, despite this proportion of missing values, shows the very late stage at presentation of our patients. Since the proportional hazards assumption was violated, we did not run multivariate survival models. However, the differences in stage distribution by regime of affiliation, with lower stage at diagnosis of breast cancer in the contributive and special regimes as well as privately insured women, explains the differences between survival curves for breast cancer. Likewise, for cervical cancer, the better survival of the privately insured is most likely due to the earlier stage at diagnosis in this group of patients.

It is important to have a baseline idea of hospital-based cancer survival, to evaluate tendencies and be able to act when necessary. Counting with reliable data on cancer occurrence, stage and survival is necessary for effective cancer control, at local and national level. 

## Conclusion

Breast cancer and cervical cancer have, for international standards, a poor survival, and this survival has not improved over time. The late stage at diagnosis undoubtedly plays an important role in these relatively poor results and could be improved through changes in the early detection programs offered in Colombia. The statistically significant differences in clinical stage by type of affiliation to the social security system is reflected in the survival rates and shows the enormous potential for improvement in access to early detection, diagnosis and treatment of these cancer types. 
