# Supplementary material for: Breast and cervical cancer survival at Instituto Nacional de Cancerología, Colombia
Source: Colomb Med (Cali). 2018 Mar 30;49(1):102–8. doi: 10.25100/cm.v49i1.2840 (PMC6018823; doi:10.25100/cm.v49i1.2840)
Supplement: Related texts

# Research article: Supervivencia al cáncer de mama y cervix en el Instituto Nacional de Cancerología, Colombia

## Introducción 

La carga de cáncer en las mujeres de América Latina se atribuye principalmente a los cánceres de mama y cuello uterino ^1^^-^^3^. En 2012, en Latinoamérica, las tasas de incidencia estandarizadas por edad de cáncer de mama alcanzaron niveles entre 40-65 por 100,000 años-mujer. Las tasas estandarizadas por edad de mortalidad por cáncer de mama en Colombia aumentaron sustancialmente: de 6.9 a mediados de los años 80 a 10.8 en 2012 ^4^^-^^6^. Las tasas de mortalidad estandarizadas por edad para cáncer de cuello uterino disminuyeron de 13.2 en 1984-1988 a 8.7 en 2013 ^6^.

El Instituto Nacional de Cancerología de Colombia (INC) estimó que en el período 2007-2011, se diagnosticaron alrededor de 7,600 casos nuevos de cáncer de mama al año, con 2,226 muertes anuales. En el mismo período, hubo 4,462 nuevos casos de cáncer de cuello uterino y 1,861 muertes. Ambos tipos de cáncer muestran fuertes variaciones geográficas entre las distintas partes del país, con cáncer de mama más frecuente en las ciudades y áreas urbanizadas, y cáncer de cuello uterino en las áreas remotas ^7^.

El pronóstico de estos dos tipos de cáncer depende de las características sociodemográficas, pero aún más del estadio clínico al momento del diagnóstico, las opciones terapéuticas disponibles y la eficacia del sistema para permitir el acceso a la atención. En general, la supervivencia del cáncer mejora con el Índice de Desarrollo Humano (IDH) de los países o regiones, probablemente a través de un mejor acceso a tratamientos eficientes y una detección potencialmente temprana. Colombia se clasifica actualmente como un país con un IDH alto (0.720) ^8^. Desde 2003, la cobertura del sistema de aseguramiento en salud obligatorio y universal de Colombia aumentó sustancialmente. Este sistema consiste principalmente en dos regímenes diferentes, cada uno de los cuales cubre poco menos del 50% de la población, asignados en función de los ingresos: el régimen contributivo, que cubre a los trabajadores y sus familias con un ingreso superior al límite y financiado a través de la nómina y las contribuciones del empleador y el régimen subsidiado, que cubre a los identificados como "pobres". Además, alrededor del 5% de la población, los trabajadores de la industria del petróleo, los maestros, el ejército y la policía están afiliados a regímenes "especiales" y "excepcionales"; y un grupo restante de la población no está cubierto por el sistema (representó el 2.6% en 2015, según el Ministerio de Salud) ^9^^-^^10^. Los paquetes y métodos de aseguramiento son similares, pero no las entidades de aseguramiento que proveen la atención dentro de cada régimen.

Existen pocos datos sobre la supervivencia de estos dos tipos de cáncer en las poblaciones de América Latina; los datos basados en la población no muestran la supervivencia por estadio clínico ^11^^,^^12^. Las tendencias de la supervivencia en entornos hospitalarios también son escasas ^13^, y la mayoría de los informes existentes apuntan a determinar la eficacia de las diferentes opciones terapéuticas. Es así que el INC Colombia diseñó un sistema de vigilancia de supervivencia, basado en vínculos con bases de datos gubernamentales, para producir estimaciones de supervivencia global comparables de sus pacientes sobre una base anual, con el objetivo de evaluar cambios en el pronóstico a lo largo del tiempo y contribuir a la mejora de la calidad del tratamiento del cáncer dentro de la institución y, a través de la comparación de datos, a nivel nacional.

En este manuscrito se comparan las características demográficas y clínicas de las pacientes con cáncer de mama y cuello uterino tratados en el INC en los años 2007, 2010 y 2012 y se informa la supervivencia global a dos años estimada por edad, estadio clínico y tipo de afiliación al sistema de seguridad social.

## Materiales y Métodos

Se seleccionaron los casos en mujeres con cáncer invasivo de mama (C50) y de cuello uterino (C53) tratados por primera vez en el INC - Colombia en los años 2007, 2010 y 2012 a partir del registro de cáncer hospitalario del INC ^14^. Solo se consideró el primer cáncer primario invasivo para cada tipo de cáncer, ya que la probabilidad de supervivencia de las pacientes con primarios anteriores pudo verse alterada. Los casos registrados en cada año se consideraron cohortes fijas. La información del registro hospitalario se verificó y completó utilizando la información de las historias clínicas y se vinculó con la base de datos de mortalidad hospitalaria y con fuentes de información gubernamentales, como el Registro Nacional Civil (RNEC), para determinar el estado vital a diciembre 31 de 2014 y la fecha de fallecimiento en aquellas pacientes fallecidos fuera del INC. Esto fue necesario ya que la legislación colombiana no permite la vinculación directa entre nuestras bases de datos de pacientes y la causa y fecha del registro de defunciones nacional; si se tiene el número de identificación personal, es posible verificar el estado vital y el registro de las muertes en RNEC. Se calculó la supervivencia global a dos años para las cohortes de mujeres que ingresaron a INC en 2007, 2010 y 2012. Se definió como fecha de inicio de seguimiento la fecha de ingreso al INC. La fecha de fallecimiento se especificó de acuerdo con el certificado de defunción en caso de que este certificado estuviera disponible. Para aquellas pacientes reportadas como fallecidas en el RNEC pero sin una fecha específica de la muerte, se determinó la fecha esperada de la muerte como la fecha de reporte de los fallecimientos en RNEC menos un factor de corrección. Esta corrección se calculó sobre la base de datos de pacientes fallecidas con certificados de defunción disponibles, donde se comparó la fecha real de fallecimiento con la fecha de notificación en RNEC; la mediana de la diferencia entre estas fechas se restó de la fecha de registro de la muerte en la RNEC para obtener la fecha esperada de la muerte ^15^. Esta mediana (en días) disminuyó con el tiempo (para cáncer de mama fue de 148 días en 2007, 66 en 2010 y 25 en 2012; el número correspondiente de días para cáncer de cuello uterino fue de 184, 114 y 39 días), lo que indica mejora sustancial en los sistemas de notificación. Los pasos detallados para determinar la fecha del último contacto o la fecha de fallecimiento se describieron en forma detallada en una publicación ^15^. Por la complejidad del proceso, se describe con más detalle a continuación:

a) Para las pacientes que fallecieron en el INC se asignó la fecha exacta de la muerte conocida.

b) Para pacientes con estado vital desconocido, se utilizó el número de identificación personal colombiano (cédula) para verificar el estado vital en las bases de datos del RNEC. La RNEC tiene información sobre las personas que han fallecido. Si las pacientes no aparecieron como "fallecidas" en ninguna de las fuentes de datos de la RNEC, se asignó como última fecha de seguimiento el 31 de diciembre de 2014.

c) Para los casos reportados como fallecidos en RNEC pero sin información del certificado de defunción, la fecha de muerte se estimó con base en la fecha de notificación o registro de la muerte en la RNEC, corregida por la diferencia mediana entre la fecha de defunción y la fecha de notificación de muerte en RNEC, como se describió anteriormente ^15^. En caso de obtener los tiempos de supervivencia negativos, se asignó la fecha informada en la RNEC como fecha de muerte. Este procedimiento generó la variable: fecha calculada de muerte. 

d) Para aquellos casos fallecidos según RNEC pero con solo el año de fallecimiento conocido (sin mes o día disponible en RNEC), se asignó el 30 de junio del año provisto como fecha de fallecimiento para las pacientes con fecha de entrada en el primer semestre del año y el 31 de diciembre si las pacientes ingresaron a INC en el segundo semestre.

 e) Para aquellos casos en los que ninguno de estos métodos se logró aplicar, o que no se identificaron en las bases de datos mencionadas, la última fecha de seguimiento se asignó como la fecha de la última visita a la institución de acuerdo con la historia clínica de INC.

### Análisis estadístico

Se usó como fecha índice para el cálculo del tiempo de supervivencia la fecha de ingreso a la institución. Se calculó la variable “tiempo de observación” a partir de la fecha índice y la fecha del último contacto o muerte.

Para evaluar las diferencias en la distribución del estadio clínico por tipo de afiliación al sistema de seguridad social colombiano, se realizó la prueba exacta de Fisher. El tiempo de supervivencia se calculó como la diferencia entre la fecha de cierre del seguimiento (31 de diciembre de 2014), la fecha del último contacto o la fecha calculada de fallecimiento y la fecha de ingreso al INC. Se realizó el análisis de Kaplan-Meier para obtener la probabilidad acumulada de supervivencia global a 24 meses y se compararon las funciones de supervivencia en las variables que tenían más de una categoría con la prueba de rango logarítmico. 

Se realizaron análisis univariados para el año de ingreso, edad en dos categorías (<50 y ≥50 años), estadio clínico y tipo de afiliación al sistema de seguridad social al momento de ingresar al INC. Por la violación del supuesto de hazards proporcionales para las variables año, grupo de edad y tipo de afiliación al sistema de seguridad social no se informaron los resultados de los modelos multivariados de riesgos proporcionales de Cox. Todos los datos fueron analizados en SPSS v19.

## Resultados

### Datos demográficos y características clínicas de las pacientes

Se analizaron un total de 1,928 pacientes con cáncer de mama y 1,189 con cáncer de cuello uterino. La Tabla 1 muestra las características por tipo de cáncer y cohorte; la distribución del cáncer de mama y de cuello uterino cambió a lo largo del tiempo. Los casos de cáncer de mama se concentraron en el grupo de edad de 45-54 años y cáncer de cuello uterino en el grupo de 15-44 años. El porcentaje de pacientes no afiliadas al sistema de seguridad social disminuyó entre 2007 y 2012 para ambos tipos de cáncer. Hubo una proporción importante de casos sin información de estado clínico (19.7% para cáncer de mama, 15.0% para cáncer de cuello uterino); El 100% de los casos tenían un diagnóstico confirmado histológicamente.

**Tabla 1:** Características demográficas y diagnósticas de la población de estudio por cohortes.

Characteristics	Breast cancer								Cervical cancer							
	n	%	2007		2010		2012		n	%	2007		2010		2012	
Total number	1,928	100	622	32.3	632	32.8	674	35.0	1,189	100	387	32.5	474	39.9	328	27.6
Age (years)	*** ***	*** ***														
0 - 14	0	0.0	0	0.0	0	0.0	0	0.0	0	0.0	0	0.0	0	0.0	0	0.0
15 - 44	344	17.8	118	19.0	102	16.1	124	18.4	400	33.6	119	30.7	167	35.2	114	34.8
45 - 54	609	31.6	189	30.4	199	31.5	221	32.8	293	24.6	102	26.4	97	20.5	94	28.7
55 - 64	493	25.6	158	25.4	165	26.1	170	25.2	262	22.0	86	22.2	116	24.5	60	18.3
> 65	482	25.0	157	25.2	166	26.3	159	23.6	234	19.7	80	20.7	94	19.8	60	18.3
Social Security Scheme	*** ***	*** ***														
Contributive	764	39.6	228	36.7	236	37.3	300	44.5	286	24.1	86	22.2	85	17.9	115	35.1
Subsidized	525	27.2	119	19.1	219	34.7	187	27.7	573	48.2	154	39.8	289	61.0	130	39.6
Special	168	8.7	50	8.0	50	7.9	68	10.1	45	3.8	9	2.3	9	1.9	27	8.2
Particular	256	13.3	90	14.5	80	12.7	86	12.8	98	8.2	30	7.8	39	8.2	29	8.8
Uninsured	215	11.2	135	21.7	47	7.4	33	4.9	187	15.7	108	27.9	52	11.0	27	8.2
Clinical stage	*** ***	*** ***														
I	111	5.8	26	4.2	43	6.8	42	6.2	289	24.3	106	27.4	112	23.6	71	21.6
II	545	28.3	168	27.0	196	31.0	181	26.9	250	21.0	92	23.8	104	21.9	54	16.5
III	751	39.0	259	41.6	248	39.2	244	36.2	418	35.2	121	31.3	173	36.5	124	37.8
IV	141	7.3	34	5.5	54	8.5	53	7.9	54	4.5	14	3.6	21	4.4	19	5.8
No information	380	19.7	135	21.7	91	14.4	154	22.8	178	15.0	54	14.0	64	13.5	60	18.3

### Supervivencia global

La supervivencia global (SG) a dos años para los cánceres de mama y cuello uterino no varió entre las diferentes cohortes, como se muestra en la Figura 1. 

**Figura 1:** Comparación de la supervivencia global estimada a dos años para cáncer de mama y cuello uterino, para las tres cohortes

**Mama (CM)**

Los 1,928 pacientes analizadas tuvieron una mediana de edad de 55 años (rango 17-99) al ingreso en el INC. La mayoría (66.0%) de las pacientes tenía más de 50 años y la mayor proporción (39.6%) estaban afiliados al régimen "contributivo" del sistema de seguridad social. Aproximadamente la mitad de las pacientes tenía cáncer de mama en estadio III-IV al ingresar al INC. La distribución del estadio clínico difirió sustancialmente y estadísticamente entre los regímenes, con alrededor del 60.0% de las mujeres en los grupo subsidiado y no asegurados en las etapas III-IV versus 42.0% en el contributivo y alrededor del 30% en el régimen especial y el grupo de pacientes particulares (Tabla 2).

**Tabla 2:** Distribución del estadio clínico por tipo de afiliación al sistema de seguridad social .

Estadio clínico	CONTRIBUTIVO	SUBSIDIADO	NO ASEGURADO	ESPECIAL	PARTICULAR
	%	%	%	%	%
Cáncer de mama					
I	8.2	2.1	2.3	12.5	4.3
II	32.1	25.1	22.3	35.8	23.4
III	35.0	51.1	48.8	29.2	24.2
IV	6.9	8.6	13.5	3.0	3.5
Sin información	17.8	13.1	13.0	19.6	44.5
Cáncer de cuello uterino					
I	26.9	25.2	20.3	31.1	16.3
II	21.7	21.1	21.4	20.0	18.3
III	33.5	35.7	42.8	24.4	26.5
IV	5.2	4.4	5.4	2.2	3.0
Sin información	12.6	13.6	10.2	22.2	35.7

Valores de P para las diferencias en la distribución del estadio por régimen: cáncer de mama: *p*= 0.01034, cáncer de cuello uterino *p* <0.005

A los 24 meses de seguimiento, 393 mujeres (20.4%) habían muerto, los 1,535 casos restantes fueron censurados en el seguimiento. La SG de dos años para el cáncer de mama fue del 79.6%, con claras diferencias en la supervivencia según los tipos de afiliación al sistema de seguridad social, siendo más alta en el régimen "especial" (93.4%) (Prueba de rango log 48.9, *p* <0.001). Pocas pacientes murieron en el primer mes de seguimiento (1.9%). La SG de dos años de las pacientes para el estadio clínico I fue muy alta (98.2%), disminuyendo a 36.2% en las de estadio IV. No se encontraron cambios significativos para el año de ingreso al INC o la edad (Tabla 3, Fig. 2).

**Cuello uterino (CCU)**

**Figura 2:** Función de supervivencia a dos años (Kaplan - Meier) por estadio clínico y seguridad social, para cáncer de mama (a y b) y de cuello uterino (c y d).

**Tabla 3:** Estimaciones univariadas de supervivencia global de cáncer de mama y cáncer de cuello uterino, por cohortes.

	Cáncer de mama						Cáncer de cuello uterino					
Características	n	%	Número de muertes *	Supervivencia**	IC 95%	Prueba de rango logarítmico	n	%	Número de muertes*	Supervivencia**	IC 95%	Prueba de rango logarítmico
Total	1,928		393	796	77.8-81.4	N.A.	1,189		435	633	60.6 - 66.0	N.A.
												
Año de ingreso al INC												
2007	622	323	122	804	77.3-83.5	X^2^= 4.1	387	325	135	650	60.3 - 69.7	X^2^= 3.7
2010	632	328	142	774	74.1-80.7	*p=* 0.127	474	399	177	626	(58.3 - 66.9	*p=* 0.161
2012	674	350	129	808	77.9-83.7	* *	328	276	123	623	(57.0 - 67.6	* *
Edad (años)						* *						* *
<50	655	340	138	789	75.8-82.0	X^2^= 0.5	539	453	173	678	(63.9 - 71.7	X^2^= 17.6
≥ 50	1,273	660	255	800	77.8-82.2	*p=* 0.481	650	547	262	595	(55.8 - 63.2	*p=* 0.000
Tipo de seguridad social												
Contributivo	764	396	147	808	78.1-83.5	*X* ^*2*^ *= 48.9*	286	241	102	642	(58.7 - 69.7	X^2^=6.0
Subsidiado	525	272	131	750	71.3-78,7	*p=* 0.000	573	482	201	648	(60.9 - 68.7	*p=* 0.202
Especial	168	87	11	934	(89,7 - 97,1)	* *	45	38	16	644	50.5 - 78.3)	* *
Particular	256	133	52	796	74.7 - 84.5	* *	98	82	46	526	(42.6 - 62.6	* *
No asegurado	215	112	52	758	70.1 - 81.5	* *	187	157	70	624	(55.3 - 69.5	* *
Estadio Clínico												
I	111	58	2	982	95.6 - 100.7	X^2^= 404.9	289	243	28	903	87.0 - 93.6	X^2^= 229.5
II	545	283	30	945	92.5 - 96.5	*p=* 0.000	250	210	61	756	70.3 - 80.9	*p=* 0.000
III	751	390	171	772	74.3 - 80.1	* *	418	352	217	476	42.7 - 52.5	* *
IV	141	73	90	362	28.4 - 44.0	* *	54	45	41	226	11.4 - 33.8	
Sin información	380	197	100	736	69.1 - 78.1		178	15	88	50.6	43.3 - 57.8	

*Número de muertes a dos años de seguimiento

**Probabilidad de supervivencia a 2 años

N.A. = no aplica

La edad media de las 1,189 mujeres con cáncer de cuello uterino fue de 51 años (rango 19-92). La mayoría tenía más de 50 años, casi la mitad (48.2%) estaban afiliadas al régimen subsidiado del sistema de seguridad social, y cerca del 50% de las pacientes ingresaron a INC en estadios I o II. La distribución por estadio clínico fue similar entre los regímenes, entre 40 y 50% de las mujeres diagnosticadas en estadios I-II, con excepción de las mujeres que ingresaron como particulares (34.6%), las cuales tenían un estadio clínico significativamente mejor al ingreso (Tabla 2). Al final de los dos años de seguimiento, 435 (36.6%) habían muerto, los 754 casos restantes fueron censurados. La probabilidad de sobrevivir dos años fue del 63.3%, con una mejor supervivencia para las mujeres con edades más tempranas (67.8%). Durante el primer mes de seguimiento, el 2.3% de las pacientes fallecieron. Hubo un fuerte gradiente en la presentación de la supervivencia por estadio clínico, entre el 90.3% en el I y el 22.6% en el IV. No se observaron diferencias en la supervivencia por año de ingreso o tipo de seguridad social (Tabla 3, Fig. 2).

## Discusión

Estos son los primeros resultados del proyecto de vigilancia de supervivencia general del INC; mostrando una supervivencia general estable a 2 años para cáncer de mama y cáncer cervical con las diferencias esperadas por estadio clínico y diferencias importantes por tipo de afiliación a la seguridad social para cáncer de mama entre 2007 y 2012. La asociación conocida entre indicadores socioeconómicos y cáncer de mama y cuello uterino se reflejan en la mayor proporción de pacientes con cáncer de mama en los regímenes contributivo y especial, en comparación con la mayoría del cáncer de cuello uterino que está afiliado al régimen subsidiado, mientras que la distribución de la población de personas afiliadas no difiere mucho entre los dos regímenes ^16^.

Existen pocas publicaciones sobre la supervivencia del cáncer de mama y de cuello uterino en Colombia y América Latina ^11^^,^^12^^,^^17^. La supervivencia global informada de dos años para mama (80%) y cáncer de cuello uterino (63%) no está lejos de los informes poblacionales de Cali (SG a 3 años: mama 77%, cérvix 63%) ^12^. Se espera que la supervivencia en un hospital especializado en cáncer sea más baja que los datos de supervivencia basados en la población, porque los centros especializados tienden a recibir pacientes "complicados" derivados de otros hospitales menos especializados.

Una limitación del estudio fue no tener una fecha de incidencia confiable, y por lo tanto se usó la fecha de ingreso al INC. El tiempo de supervivencia calculado desde la fecha del diagnóstico es ciertamente más alto que los datos de supervivencia reportados, aunque no se puede cuantificar cuanto más alto: algunos pacientes acuden para su tratamiento inicial al INC; su fecha de ingreso será cercana a la fecha del diagnóstico; otros aparecen cuando el tratamiento inicial ha fallado o cuando tienen una recurrencia, a veces mucho después de su diagnóstico inicial. Teniendo en cuenta esto, las tasas de supervivencia reportadas para cáncer de cuello uterino son relativamente similares a las estimaciones europeas, inferiores a las de EE. UU., pero más altas que las de Brasil ^11^^,^^12^. La supervivencia del cáncer de mama es un poco menor que los informes de estudios basados en los EE. UU., indudablemente relacionados con una etapa relativamente tardía al ingreso en nuestras pacientes, con un 46% de mujeres con enfermedad en estadio III / IV.

La calidad del registro de defunciones en Colombia ha mejorado sustancialmente en los últimos años ^18^, lo que limita, pero no elimina, la posibilidad de que algunos pacientes hayan muerto sin ser registrados. En este caso, la paciente habrá sido censurada viva en su última visita al INC, que puede tener tasas de supervivencia ligeramente sobreestimadas. La calidad mejorada también se refleja en la disminución del tiempo entre la fecha de la muerte y la notificación de la muerte en RNEC. 

La distribución por edades de nuestras pacientes fue la esperada ^19^^,^^20^. Para el pronóstico del cáncer de mama, la detección temprana es importante, así como el tiempo transcurrido entre los primeros síntomas o la prueba de detección anormal y la primera consulta (según un estudio colombiano anterior fue >1 mes en el 34,1% de las pacientes) y el tiempo transcurrido entre la primera consulta y el inicio del tratamiento (en el 69.8% de las pacientes >3 meses)^(1)^. Las pacientes con cáncer cérvico-uterino, como era de esperar, relativamente jóvenes, y una proporción sustancial (40%) presentaba enfermedad en estadio avanzado, una situación que podría haberse evitado con programas eficaces de detección temprana.

Infortunadamente, no se contó con información sobre el estadio clínico en una proporción importante de las pacientes (cáncer de mama, 19.7% y cáncer de cuello uterino, 15.0%). Sin embargo, a pesar de esta proporción de valores perdidos, las observaciones muestran un estadio clínico muy avanzado de presentación en las pacientes, alrededor del 50% de las mujeres diagnosticadas en cáncer de mama en estadio III / IV y 40% en cáncer de cuello uterino en estadio III / IV. Como se violó la suposición de hazards proporcionales, no se ejecutaron modelos de supervivencia multivariados. Sin embargo, las diferencias en la distribución por estadio clínico según el régimen de afiliación, con un estadio más bajo en el momento del diagnóstico de cáncer de mama en los regímenes contributivo y especial, así como en las mujeres que ingresaron como particulares, explican las diferencias entre las curvas de supervivencia del cáncer de mama. Del mismo modo, para el cáncer de cuello uterino, la mejor supervivencia de las que ingresaron como particulares es más probable debido al estadio más temprano en el momento del diagnóstico en este grupo de pacientes.

Es importante tener una línea de base de la supervivencia del cáncer en la institución, evaluar las tendencias y poder actuar cuando sea necesario. Para contar con un control eficaz del cáncer a nivel local y nacional, es necesario contar con datos confiables sobre la aparición del cáncer, el estadio y la supervivencia.

## Conclusión

El cáncer de mama y el cáncer de cuello uterino tienen, para los estándares internacionales, una supervivencia deficiente, y esta supervivencia no ha mejorado con el tiempo. La última etapa del diagnóstico indudablemente juega un papel importante en estos resultados relativamente pobres y podría mejorarse a través de cambios en los programas de detección temprana ofrecidos en Colombia. Las diferencias estadísticamente significativas en el estadio clínico por tipo de afiliación al sistema de seguridad social se reflejan en las tasas de supervivencia y muestran el enorme potencial de mejora en el acceso a la detección temprana, el diagnóstico y el tratamiento de estos tipos de cáncer.
